# Antifibrotic Effects of Caffeine, Curcumin and Pirfenidone in Primary Human Keratocytes

**DOI:** 10.3390/ijms24021461

**Published:** 2023-01-11

**Authors:** Delia Talpan, Sabine Salla, Nina Seidelmann, Peter Walter, Matthias Fuest

**Affiliations:** 1 Department of Ophthalmology, RWTH Aachen University, 52074 Aachen, Germany; 2 Cornea Bank Aachen, RWTH Aachen University, 52074 Aachen, Germany

**Keywords:** cornea, keratocyte, cell culture, fibrosis, scarring

## Abstract

We evaluated the small molecules (AFM) caffeine, curcumin and pirfenidone to find non-toxic concentrations reducing the transformation of activated human corneal stromal keratocytes (aCSK) to scar-inducing myofibroblasts (MYO-SF). CSK were isolated from 16 human corneas unsuitable for transplantation and expanded for three passages in control medium (0.5% FBS). Then, aCSK were exposed to concentrations of caffeine of 0–500 μM, curcumin of 0–200 μM, pirfenidone of 0–2.2 nM and the profibrotic cytokine TGF-β1 (10 ng/mL) for 48 h. Alterations in viability and gene expression were evaluated by cell viability staining (FDA/PI), real-time polymerase chain reaction (RT-PCR) and immunocytochemistry. We found that all AFMs reduced cell counts at high concentrations. The highest concentrations with no toxic effect were 100 µM of caffeine, 20 µM of curcumin and 1.1 nM of pirfenidone. The addition of TGF-β1 to the control medium effectively transformed aCSK into myofibroblasts (MYO-SF), indicated by a 10-fold increase in α-smooth muscle actin (SMA) expression, a 39% decrease in lumican (LUM) expression and a 98% decrease in ALDH3A1 expression (*p* < 0.001). The concentrations of 100 µM of caffeine, 20/50 µM of curcumin and 1.1 nM of pirfenidone each significantly reduced SMA expression under TGF-β1 stimulation (*p* ≤ 0.024). LUM and ALDH3A1 expression remained low under TGF-β1 stimulation, independently of AFM supplementation. Immunocytochemistry showed that 100 µM of caffeine, 20 µM of curcumin and 1.1 nM of pirfenidone reduce the conversion rate of aCSK to SMA^+^ MYO-SF. In conclusion, in aCSK, 100 µM of caffeine, 20 µM of curcumin and 1.1 nM of pirfenidone significantly reduced SMA expression and MYO-SF conversion under TGF-β1 stimulation, with no influence on cell counts. However, the AFMs were unable to protect aCSK from characteristic marker loss.

## 1. Introduction

Corneal stromal scarring remains a major cause of blindness worldwide, with limited treatment options [1,2]. Damage to the mammalian cornea causes a rapid cellular reaction, which attempts to heal the wound. Disruption of the epithelial layer and basement membrane and injury to corneal stromal keratocytes (CSK) release a range of cytokines, including the platelet-derived growth factor (PDGF) and transforming growth factor β (TGF-β), the latter of which is probably the strongest pro-fibrotic agent [3,4].

Human CSK are quiescent in vivo and typically express stromal crystalline proteins, including aldehyde dehydrogenases (ALDH, type 1A1 and 3A1), a-enolase, lactic dehydrogenase and transketolase, contributing to corneal transparency [4,5]. They also synthesize and deposit collagens and keratan sulfate proteoglycans (lumican, keratocan and mimecan), as well as enzymes (such as collagenases), to degrade old matrix proteins for stromal matrix homeostasis. In injured corneas, human CSK exhibit a rapid loss of the keratocyte phenotype [4,6,7]. The “activated” keratocytes (aCSK) differentiate into stromal fibroblasts (SF) and display a different gene expression profile [8,9]. Under the influence of TGF-β, they further transform into myofibroblasts (MYO-SF), expressing α-smooth muscle actin (SMA), which grants them contractile properties [4,10].

Though undoubtedly useful for the goal of shrinking the injury site, wound contraction in the cornea disturbs the shape and curvature of this critical optic and its ability to precisely focus light onto the retina. Loss of transparency, largely in the form of haze during corneal wound healing, results from several factors: (1) infiltration of the cornea by inflammatory cells, (2) SMA-positive MYO-SF, which are less transparent than quiescent CSK, likely due to their decreased crystallin synthesis [11], and (3) MYO-SF laying down an extracellular matrix (ECM) that is both differently organized and composed of molecules which are neither part of the normal stromal ECM nor conducive to good corneal transparency [5,11].

Expanding human CSK is challenging and this has been a limiting factor in their application in cellular research, corneal tissue engineering and cell therapy [4,12]. Medium supplementation with low levels (0.5%) of fetal bovine serum (FBS) or human platelet lysate (hPL) activates CSK (aCSK) and promotes cell division, but also leads to a more fibroblastic phenotype and a reduction in the characteristic CSK markers [12,13]. When aCSK are cultured in a serum-free medium for 7–21 days, they can regain their bona fide CSK morphology and phenotype [12]. SF are usually induced in vitro by adding ≥5% FBS to the medium. However, similar to in vivo conditions, the transformation is believed to be irreversible, even when SF are switched to a serum-free medium [4,12]. The differences in marker expression and phenotype between CSK, aCSK, SF and MYO-SF have been extensively investigated by Yam et al. [4,12].

Clinically, attempts to control corneal scarring have mostly involved the use of steroids or mitomycin C (MMC) [14]. While effective in decreasing MYO-SF-differentiation and haze, these two compounds exhibit significant side-effects and, in the case of MMC, toxicity and DNA damage to CSK and endothelial cells, which could bear long-term negative consequences for ocular health [15]. Experimentally, ocular application of antibodies against TGF-β after excimer laser ablation of the corneal surface reduced MYO-SF-differentiation, migration and corneal reflectivity (haze) in both rabbits [16] and cats [17]. However, epithelial healing was slowed and, in cases of application for longer than 3 days, stromal regeneration was also blocked [17].

Consequently, there is an urgent need for antifibrotic agents that effectively impede CSK from MYO-SF conversion and corneal scarring, while exerting as little toxicity as possible, in vivo and in vitro.

In recent years, efforts were undertaken to investigate whether small antifibrotic molecules (AFM), capable of manipulating intracellular signaling downstream of TGF-β receptor activation, might represent a better alternative to steroids, MMC and topical application of anti-TGF-β antibodies to the eye [18,19,20]. In this study, we investigated a potential antifibrotic effect of the AFMs caffeine, curcumin and pirfenidone, on primary human aCSK for future in vivo and in vitro use.

Caffeine inhibited TGF-β activation in lung epithelial cells and interrupted lung fibroblast responses to TGF-β in concentrations between 50 µM and 100 µM [21].

Curcumin at concentrations of 5–20 μmol/L significantly inhibited UVB-induced secretion of IL-6 and IL-8 by human limbus epithelial cells in a dose-dependent manner, while curcumin alone did not affect the secretion of IL-6 and IL-8 [22].

Jiang et al. showed that pirfenidone inhibited human umbilical vein endothelial cell (HUVEC) viability at concentrations of ≥300 μg/mL, but eye drops containing 1000 μg/mL of pirfenidone administrated four times per day for 14 days reduced corneal edema, promoted corneal wound healing and inhibited neovascularization formation in a rat alkali burn model [23].

However, the effects of caffeine, curcumin and pirfenidone on primary human aCSK have not yet been investigated. Hence, we investigated their antifibrotic potential in concentrations of caffeine of 0–500 μM, curcumin of 0–200 μM and pirfenidone of 0–2.2 nM (0–400 µg/mL).

## 2. Results

### 2.1. Cell Number Analysis

To evaluate the potential toxic effects of the AFMs, aCSK were incubated in different media (Table 1) for 48 h, then stained using 5% fluorescein diacetate (FDA) and 5% propidium iodide (PI) and viable green cells were counted and compared.

**Figure 1:**
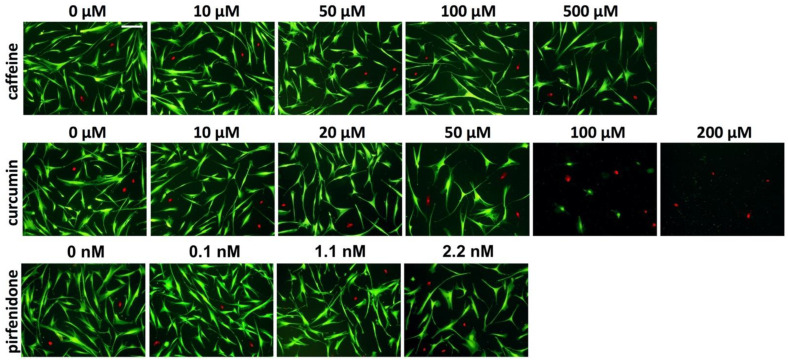
Activated corneal stromal keratocytes (aCSK) incubated in media for 48 h containing the indicated concentrations of the antifibrotic molecules (AFM) caffeine, curcumin and pirfenidone. Green cells are alive, red cells are dead. Scale bar: 200 µm.

**Figure 2:**
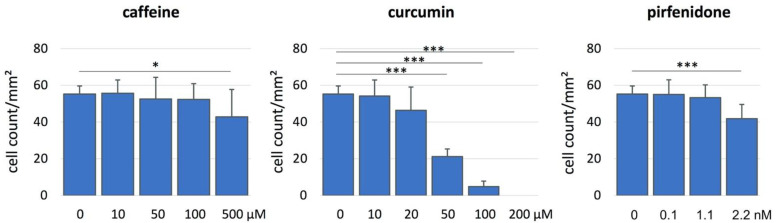
Number of activated corneal stromal keratocytes (aCSK) per mm^2^ after incubation for 48 h in media containing the indicated concentrations of the antifibrotic molecules (AFM) caffeine, curcumin and pirfenidone. Significant differences to the control (0 M) are indicated by * *p* ≤ 0.05, *** *p* ≤ 0.001.

Following 48 h of incubation, caffeine showed significantly reduced aCSK counts in comparison to the control in the highest tested concentration of 500 µM (Table 1, Figure 1 and Figure 2; *p* = 0.027).

In curcumin, a significant cell reduction effect was seen at concentrations of 50 µM and above, in comparison to the control (*p* < 0.001; Table 1, Figure 1 and Figure 2). aCSK cell numbers were further reduced at 100 µM of curcumin (cell count/mm^2^: 4.8 ± 3.0; *p* < 0.001) and 200 µM of curcumin (cell count/mm^2^: 0.0; *p* < 0.001).

In pirfenidone, the highest tested concentration of 2.2 nM (400 µg/mL) significantly reduced the aCSK cell count (*p* < 0.001; Table 1, Figure 1), in comparison to the control group.

The addition of 10 ng/mL of TGF-β1 to the medium did not influence the cell count at different AFM concentrations (*p* > 0.6; Table 1).

Consequently, the highest concentrations with no toxic effect on aCSK after 48 h of incubation were 100 of µM caffeine, 20 of µM curcumin and 1.1 nM of (200 µg/mL) pirfenidone.

### 2.2. Effects of TGF-β1 on aCSK

**Figure 3:**
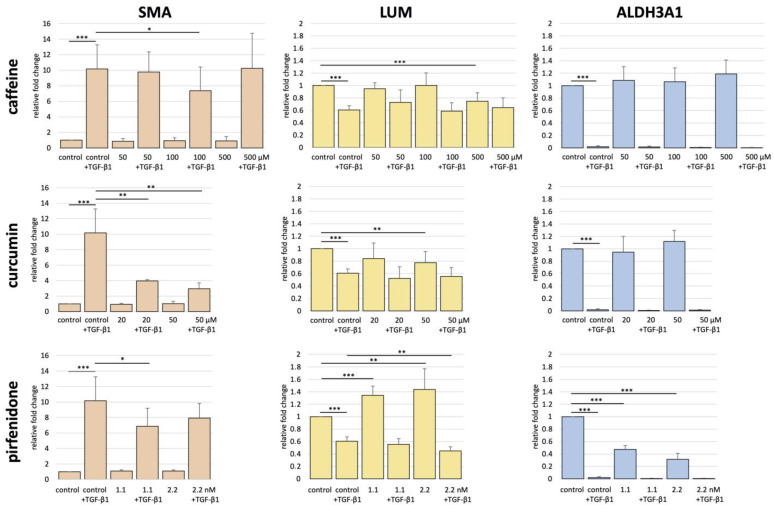
Comparison of gene expression by real-time polymerase chain reaction (RT-PCR) for α-smooth muscle actin (SMA), lumican (LUM) and aldehyde dehydrogenase family 3 member A1 (ALDH3A1) in activated corneal stromal keratocytes (aCSK) after incubation for 48 h in media containing the indicated concentrations of the antifibrotic molecules (AFM) caffeine, curcumin and pirfenidone, with and without the profibrotic cytokine 10 ng/mL TGF-β1. Relative fold changes were calculated in comparison to the control group. Significant differences between groups are indicated by * *p* ≤ 0.05, ** *p* ≤ 0.01 and *** *p* ≤ 0.001.

TGF-β1 addition to the control medium led to a 10-fold increase in SMA expression (*p* < 0.001, Figure 3, Table 1), a 39% decrease in LUM expression (*p* < 0.001) and a 98% decrease in ALDH3A1 expression (*p* < 0.001) in aCSK after 48 h of incubation.

### 2.3. Effects of Caffeine on aCSK

aCSK incubated in caffeine 100 µM + TGF-β1 showed a significantly lower SMA expression compared to the control + TGF-β1 group (*p* = 0.015, Table 1, Figure 3). This effect could not be replicated in 500 µM + TGF-β1 in comparison to control + TGF-β1 (*p* = 0.49).

LUM levels of aCSK incubated in 50 and 100 µM caffeine media did not significantly differ from control aCSK. Caffeine 500 µM aCSK showed a significant decrease in LUM expression compared to aCSK incubated in control media (*p* = 0.001, Table 1, Figure 3). No significant differences in LUM expression were seen between aCSK incubated in 50, 100 or 500 µM caffeine + TGF-β1 in comparison to control + TGF-β1 aCSK.

ALDH3A1 expression levels did not differ between 50, 100 and 500 µM caffeine aCSK in comparison to control aCSK (Table 1). No significant differences in ALDH3A1 expression were found between 50 µM + TGF-β1, 100 µM + TGF-β1, 500 µM + TGF-β1 and control + TGF-β1 media.

### 2.4. Effects of Curcumin on aCSK 

A highly significant decrease in SMA expression was seen when comparing curcumin 20 µM and 50 µM + TGF-β1 to control + TGF-β1 (*p* = 0.009 and *p* = 0.002, Table 1, Figure 3).

Curcumin 50 µM showed a significant decrease in LUM expression compared to control aCSK (*p* = 0.009, Table 1, Figure 3). No significant changes in LUM expression were seen between aCSK incubated in curcumin 20 or 50 µM + TGF-β1 media and control + TGF-β1 aCSK.

ALDH3A1 expression levels did not differ between 20 or 50 µM curcumin aCSK, in comparison to control aCSK (Table 1). All groups incubated in the media containing TGF-β1 had low to no ALDH3A1 expression, regardless of curcumin supplementation.

### 2.5. Effects of Pirfenidone on aCSK 

The incubation of aCSK in pirfenidone 1.1 or 2.2 nM medium led to no significant difference in SMA expression compared to control aCSK. SMA expression levels were significantly lower in pirfenidone 1.1 nM + TGF-β1 compared to control + TGF-β1 (*p* = 0.024, Table 1, Figure 3). The SMA expression in pirfenidone 2.2 nM + TGF-β1 did not differ from control + TGF-β1 (*p* = 0.1).

aCSK incubated in pirfenidone 1.1 or 2.2 nM media showed a significant increase in LUM compared to control aCSK (*p* < 0.0001 and *p* = 0.009, Table 1, Figure 3). A significant decrease in LUM expression was detected when comparing pirfenidone 2.2 nM + TGF-β1 to control + TGF-β1 (*p* = 0.006, Table 1, Figure 3).

A significant decrease in ALDH3A1 expression was found when comparing pirfenidone 1.1 or 2.2 nM to control (*p* < 0.001 and *p* < 0.009, Table 1, Figure 3). All groups incubated in media containing TGF-β1 showed low to no ALDH3A1 expression, regardless of pirfenidone supplementation.

### 2.6. Immunocytochemistry 

**Figure 4:**
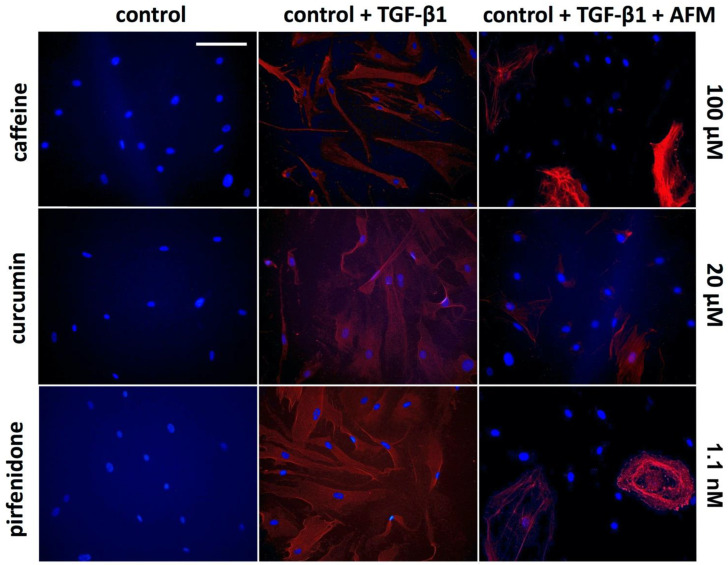
Immunhistochemistry for α-smooth muscle actin (SMA, red) and 4′,6-diamidino-2-phenylindole (DAPI, blue) in activated corneal stromal keratocytes (aCSK) after incubation for 48 h in media containing the indicated concentrations of the antifibrotic molecules (AFM) caffeine, curcumin and pirfenidone, with and without the profibrotic cytokine 10 ng/mL TGF-β1. Scale bar: 100 µm.

Immunocytochemistry staining showed no SMA expression in control aCSK (Figure 4). Following the addition of 10 ng/mL of TGF-β1 to control aCSK, almost all cells converted to SMA expressing MYO-SF. The addition of 100 µM of caffeine, 20 of µM curcumin and 1.1 nM of pirfenidone reduced the conversion rate of aCSK to SMA^+^ MYO-SF.

## 3. Discussion

In this study, we investigated the antifibrotic effect of caffeine, curcumin and pirfenidone on cultured primary human aCSK. We found that, in high concentrations, the AFMs exerted cytotoxic effects. The highest concentrations with no toxic effect on aCSK after 48 h of incubation were 100 µM of caffeine, 20 µM of curcumin and 1.1 nM (200 µg/mL) of pirfenidone. TGF-β1 addition to the control medium induced the conversion of aCSK to SMA^+^ MYO-SF and reduced the expression of the typical CSK markers lumican and ALDH3A1. The concentrations of 100 µM of caffeine, 20 µM of curcumin and 1.1 nM of pirfenidone each significantly reduced SMA expression and MYO-SF conversion under TGF-β1 stimulation with no influence on cell counts. However, they were unable to protect from CSK marker loss.

Stromal cellular responses such as CSK activation and apoptosis after surgical trauma have been studied in the corneas of mice [24], rats [5], rabbits [25] and cats [26], but rarely, due to the scarcity of tissue, in human corneas [27], where the responses may be attenuated just as the endothelial response to injury is [28]. In particular, CSK activation and regeneration appear to be decreased in human corneas after photorefractive keratectomy compared with, e.g., the responses in rabbits [29].

Hence, investigating the effect of caffeine, curcumin and pirfenidone on primary human aCSK is crucial in the search for a means of preventing haze and MYO-SF differentiation, while stimulating the regeneration of the corneal stroma and epithelium, and preserving or restoring normal ocular optics.

Caffeine (1,3,7-tri-methylxanthine) is one of the most consumed food additives worldwide and has wide-ranging pharmacological activities including effects on the central nervous, cardiovascular and respiratory systems [21]. It can act as an antagonist of adenosine receptors, an inhibitor of phosphodiesterases and an activator of ryanodine receptors. Caffeine is similar in structure and function to theophylline and can improve lung function in asthmatics by inducing bronchodilation [30]. In recent years, caffeine has been shown to exhibit anti-fibrotic effects in the liver. The consumption of caffeine, often in the form of coffee, is associated with reduced hepatic fibrosis in patients suffering from chronic hepatitis C virus infection [31]. In in vivo animal models of liver fibrosis, caffeine can reduce collagen deposition and collagen mRNA [32,33], and can block the expression of the profibrotic cytokine TGF-β [34]. Furthermore, caffeine can inhibit profibrotic responses in hepatic stellate cells, the key effector cell in the development of liver fibrosis [35]. In the lung, caffeine appears to exhibit its anti-fibrotic effects through distinct actions on both epithelial cells and fibroblasts, which are two of the key effector cells involved in the pathogenesis of pulmonary fibrosis. It has previously been reported that caffeine is capable of interrupting TGF-β-induced Smad signaling in a lung epithelial cancer cell line [36]. However, Tatler et al. recently showed that caffeine also abrogated profibrotic responses to TGF-β in lung fibroblasts. It inhibited basal expression of the SMA gene and reduced TGF-β-induced increases in profibrotic genes [21].

In our study, we showed, for the first time, that 100 µM of caffeine significantly reduced SMA expression and MYO-SF conversion in primary human aCSK. However, caffeine was unable to protect from CSK marker loss.

Caffeine is not known to undergo a significant first-pass metabolism and generally reaches its peak plasma concentrations within 30–120 min of its administration. Studies have shown that serum caffeine levels from moderate coffee consumption usually range between 20 and 50 µmol/L and do not tend to exceed levels of 70 µmol/L [37]. In general, it has been noted that toxicological symptoms often begin above concentrations of 15 mg/L (≈7.7 µM, generally mild psychological side effects such as irritability and nervousness, but also potentially palpitations, nausea, tremor, perspiration and paresthesia), while a concentration of 50 mg/L (≈257 µM) is considered “toxic”. Caffeine concentrations of 80 mg/L (≈411 µM) or greater are considered lethal [38] and, accordingly, 500 µM of caffeine reduced CSK counts and did not exert an antifibrotic effect in our setup.

Consequently, the topical, rather than systemic, application of 100 µM of caffeine eye drops in a corneal scarring model could be a coherent next investigative step.

Fibrosis within the cornea tends to be dynamic, with ongoing MYO-SF generation and apoptosis. Once mature MYO-SF develop, they persist until the requisite source of TGF-β1 and/or TGF-β2 to maintain viability is sufficiently reduced by repair. Once the levels of these TGF-β isotypes drop in the stroma, then IL-1 produced by surrounding cells (CSK, SF and MYO-SF (autocrine)), unopposed by TGF-β1 or TGF-β2, triggers increased apoptosis of the MYO-SF [39]. In our experimental setup, the strong stimulation by 10 ng/mL of TGF-β1 could have protected MYO-SF from the toxic effects of 500 µM of caffeine and, consequently, increased overall SMA expression.

Interestingly, the antifibrotic caffeine did not protect aCSK from the loss of their characteristic markers (i.e., lumican, ALDH3A1) during stimulation by 10 ng/mL of TGF-β1. The fact that CSK marker expression and SMA^+^ MYO-SF conversion show different responses to fibrosis inhibition has been shown before.

Seidelmann et al. demonstrated that the expression of the MYO-SF marker SMA decreased with incremental human platelet lysate (hPL) substitution in CSK and SF, implying an antifibrotic effect, which they attributed to the basic fibroblast growth factor (bFGF) and hepatocyte growth factor (HGF) found in hPL. Nevertheless, CSK markers decreased with higher hPL substitution [13].

Similarly, Jester et al. incubated primary rabbit CSK with 10 ng/mL of bFGF and found a fibroblast-like phenotype but negative SMA immunocytochemistry after 7 days of culture [40].

Curcumin (diferuloylmethane), derived from the rhizome of *Curcuma longa* L., belongs to the polyphenols. It was isolated over 140 years ago by Vogel and was synthesized in 1913 by Lampe [41]. Curcumin has been used for thousands of years in traditional Chinese medicine and Ayurvedic medicine in Asian countries as an active ingredient of herbal remedies to treat liver diseases, rheumatoid diseases, diabetes, atherosclerosis, infectious diseases and cancer [42]. It is characterized by an anti-oxidant, anti-inflammatory, anti-mutagenic, anti-microbial and anti-cancer activity [41]. Oral curcumin substitution was shown to decrease serum concentrations of TNFα, IL-6, TGF-β and MCP-1, major mediators of inflammation in many diseases [43].

Furthermore, it also downregulates NF-κB, cyclooxygenase 2 (COX-2), the main enzyme engaged in prostaglandin production, and 5-lipoxygenase (5-LOX) [44,45].

The inhibitory effect of curcumin was also described for epidermal mouse keratinocytes [46]. Curcumin diminished the uPA levels induced by TGF-β1 in immortalized skin keratinocytes and TGF-β-induced synthesis of fibronectin, as well as inhibited TGF-β-stimulated cell migration and invasiveness [46]. In a rabbit in vivo model of suturing-induced corneal neovascularization, Kim et al. demonstrated the efficacy of topical curcumin in inhibiting angiogenesis through decreasing VEGF mRNA levels and NF-κB phosphorylation [47].

Curcumin at concentrations of 5–20 μmol/L significantly inhibited UVB-induced secretion of IL-6 and IL-8 by human limbus epithelial cells in a dose-dependent manner, while curcumin alone did not affect the secretion of IL-6 and IL-8 [22]. The upregulation of NF-κB and MAPK pathways induced by UVB treatment was significantly inhibited by curcumin, suggesting that the NF-κB and MAPK pathways are involved in the inhibitory effect of curcumin in the UVB-induced production of IL-6 and IL-8 [22]. Consequently, curcumin inhibits numerous pathways that are also involved in corneal scarring, e.g., TGF-β and MAPK.

In our study, we showed, for the first time, that 20 µM of curcumin significantly reduced SMA expression and MYO-SF conversion in primary human aCSK. However, curcumin was unable to protect from CSK marker loss.

Bolger et al. examined the pharmacokinetics following the intravenous infusion of liposomal curcumin. Healthy individuals showed a curcumin peak plasma concentration of about 2000 ng/mL (≈5.4 µM) after 2 h at a dose of 240 mg/m^2^ and of nearly 3000 ng/mL (≈8.1 µM) after 2 h [48]. In a dose-escalation and pharmacokinetic study of nanoparticle curcumin with six healthy human volunteers, Kanai et al. measured a peak plasma concentration of 275 ± 67 ng/mL (≈0.8 µM) approximately 2 h after receiving a single dose of 210 mg [49].

Several trials on healthy subjects have supported the safety and efficacy of curcumin [50]. Despite this well-established safety, some negative side effects have been reported. Seven subjects receiving 500–12,000 mg in a dose–response study and followed for 72 h experienced diarrhea, headache, rash, and yellow stool [51]. In another study, some subjects receiving 0.45 to 3.6 g/day of curcumin for one to four months reported nausea and diarrhea and an increase in serum alkaline phosphatase and lactate dehydrogenase contents [52].

In vitro data indicated a toxic effect of 15 µM of curcumin microemulsion in a hepatocellular HepG2 cell line [53].

Accordingly, we found that curcumin in concentrations higher than 20 µM showed substantial toxic effects, with hardly any aCSK left after 48 h incubation in 100 and 200 µM. The reduced lumican expression in curcumin 50 µM aCSK is most likely also an indication for toxicity.

To target corneal scarring, further investigations with topical curcumin eye drops at concentrations of ≤20 µM could be a safe approach.

Pirfenidone (5-methyl-1-phenyl-2[1H]-pyridone) is a non-peptide pharmacologic anti-fibrotic, anti-inflammatory and anti-oxidant compound that was approved by the Food and Drug Administration (FDA) for patients with idiopathic pulmonary fibrosis (IPF) [20,54]. Several preclinical studies and clinical trials have indicated that pirfenidone is effective in treating IPF and renal fibrosis. It inhibits the production of TGF-α and free radical oxygen species (ROSs), as well as reducing IL-1, IL-6, IL-8, IL-12 and TNF-α levels [55]. Pirfenidone has also been demonstrated to have significant efficacy as an inhibitor of TGF-β [56,57,58]. Although its exact mechanism of action has not yet been elucidated, pirfenidone may also have an inhibitory effect on the platelet-derived growth factor (PDGF) and connective tissue growth factor (CTGF), and promote fibroblast apoptosis via the suppression of nuclear factor-κB (NF-κB), all of which may inhibit myofibroblast proliferation [59,60,61,62,63].

In the eye, pirfenidone has been shown to inhibit the activity of human Tenon’s fibroblasts in vitro [64], and has also been tested as an adjunctive postoperative antifibrotic for strabismus surgery in rabbits [65]. In addition, it has been evaluated as a postoperative anti-scarring agent for use in glaucoma surgery in a lagomorph model, and it was recently reported to successfully inhibit TGF-β1-induced equine corneal fibrosis in vitro [66,67]. In mice, Singh et al. showed that the inhibitory effects of pirfenidone on TGF-β and PDGF are efficacious in decreasing myofibroblast formation from both keratocyte and bone marrow-derived precursors [68].

Pirfenidone nanoparticles also improved corneal wound healing and prevented corneal fibrosis in Sprague Dawley rats in vitro and in vivo [69]. In addition, stromal-wounded ex vivo canine corneas exhibited greater optical clarity when treated with pirfenidone than when placebo treated at 21 days [20].

In our study, we showed, for the first time, that 1.1 nM (200 µg/mL) of pirfenidone significantly reduced SMA expression and MYO-SF conversion in primary human aCSK. However, pirfenidone was unable to protect from CSK marker loss under TGF-β stimulation.

Pirfenidone is commercialized as an oral immediate release formulation with an administration of 801 mg/day during the first week, followed by an increase to 1602 mg/day during the second week and a subsequent dose increase to reach 2403 mg/day after 15 days of treatment. Pirfenidone use, however, is limited by the occurrence of adverse events [70]. Gastrointestinal reactions, such as nausea, dyspepsia, diarrhea, abdominal discomfort and vomiting, are frequently associated with pirfenidone administration. Anorexia, fatigue, sedation and photosensitivity have also been reported. The frequency and intensity of these responses appear to decrease with time. However, adverse events often lead to dose reductions or treatment withdrawal [71].

In a pharmacokinetic study on 28 patients with idiopathic pulmonary fibrosis and chronic hypersensitivity pneumonitis, pirfenidone showed a rapid absorption rate, reaching the maximal concentration at around 0.86 ± 0.37 h and a peak concentration of 19.81 µg/mL [71].

Jiang et al. previously demonstrated that pirfenidone inhibited human umbilical vein endothelial cell (HUVEC) viability at concentrations of ≥300 μg/mL, but eye drops containing 1000 μg/mL of pirfenidone administrated four times per day for 14 days reduced corneal edema, promoted corneal wound healing and inhibited neovascularization formation in an alkali burn rat model [23]. Consequently, to target corneal scarring, topical pirfenidone eye drop treatment appears to be the safest approach.

Interestingly, aCSK incubated in media containing 1.1 or 2.2 nM (200 or 400 µg/mL) of pirfenidone showed a significant increase in lumican expression, which could indicate a protective effect against CSK marker loss during 0.5% FBS stimulation [4]. However, the ALDH3A1 expression changed in the opposite direction, which implies more complex effects of pirfenidone on the CSK cell character.

As a limitation, in this in vitro cell culture pilot study, we investigated the antifibrotic effects of the AFMs through alterations in CSK marker and SMA expression. Nevertheless, corneal fibrosis is a complex dynamic process influenced by various genes and proteins [72,73], among them, interleukin 1 and 6, matrix metallopeptidases, collagens, fibronectin, PDGF, HGF and keratinocyte growth factor (KGF). However, the TGF-β1- and 2- dependent induction of SMA^+^ MYO-SF is an integral part of organ fibrosis, and is frequently used to screen for the antifibrotic effects of different components in the cornea and other organs in and ex vivo [21,23]. Further studies using, e.g., proteomics or genomics at different time points, could help us to better understand the specific effects of the SMA-suppressive AFMs found in this study on corneal and CSK fibrosis induction.

To summarize, we found that 100 µM of caffeine, 20 µM of curcumin and 1.1 nM of pirfenidone each significantly reduced SMA expression and MYO-SF conversion under TGF-β1 stimulation, with no influence on cell counts. However, they were unable to protect from CSK marker loss. Further studies are necessary to investigate the ideal concentrations and application modes of caffeine, curcumin and pirfenidone for the inhibition of corneal scarring, as well as their potential for CSK ex vivo expansion for tissue-engineering and research purposes.

## 4. Material and Methods

### 4.1. Isolation of CSK 

Human CSK were isolated from 16 corneas (8 donors) unsuitable for transplantation (age 73.6 ± 11.4 years, male = 50%) supplied by the Cornea Bank Aachen. CSK were isolated and cultivated as previously described [4,5,13,74]. Briefly, corneas were washed with sterile phosphate-buffered saline (PBS, 0.1 M, Merck KGaA, Darmstadt, Germany); the central button was trephined (8.0 mm diameter) and incubated with dispase II (20 mg/mL, Roche, Basel, Switzerland) for 1 h at 37 °C. The loosened corneal epithelium and endothelium were removed by scrapping. The remaining stromal tissue was then digested with collagenase I (1.5 µg/mL, Gibco, Life Technologies, Grand Island, NE, USA) in CSK basal medium for 12 h at 37 °C. Single cells were then suspended in aCSK basal medium with 0.5% FBS (Panbiotech, Aidenbach, Germany, Table 2). Cells were seeded on collagen I-coated (type I collagen, solution from rat tail, Sigma-Aldrich, St. Louis, MO, USA) culture plates (BD Biosciences, Franklin Lakes, NJ, USA). The medium was changed every 3 days. Cells were passaged 1:2 when they reached 70–80% confluence using trypsin-EDTA (0.05%, Gibco).

### 4.2. Cell Culture of aCSK 

The CSK were cultured in aCSK basal medium containing 0.5% FBS until passage 3. After 24 h, the medium was exchanged for new medium containing the according substitutes (Table 2). The basal medium group served as the control. After 48 h of culture in the according medium, the cells were harvested for further testing.

### 4.3. Cell Number Analysis

The cells were seeded at 9000 cells/1.8 cm^2^ on collagen-I-coated 24-well plates (Corning, New York, NY, USA) and incubated in different media (Table 2) for 48 h at 37 °C. Then, the media were removed and 5% fluorescein diacetate (FDA) and 5% propidium iodide (PI) in PBS (both from Sigma-Aldrich) were added for live/dead staining. Samples were imaged by fluorescence microscopy (Leica DM6000B microscope, Leica Microsystems GmbH, Wetzlar, Germany). The numbers of live (green fluorescence) and dead cells (red fluorescence) were quantified in 10 random fields per well, using the cell counter plugin for Image J (version 1.53o, Wayne Rasband, Bethesda, MD, USA) [75]. Experiments were conducted in triplicate for 6 donors.

### 4.4. Immunocytochemistry

The cells were seeded at 9000 cells/1.8 cm^2^ on collagen-I-coated glass cover slips (VWR International, Radnor, PA, USA). After 48 h of culture in different media (Table 2), the cells were fixed with neutral buffered 4% paraformaldehyde (Sigma-Aldrich). After quenching with 50 mM of ice-cold ammonium chloride (Sigma-Aldrich), the samples were washed with PBS containing 0.2% bovine serum albumin (BSA, Sigma-Aldrich) and blocked with 1% bovine serum albumin and Triton X (1 µL/mL, Sigma-Aldrich), followed by incubation with the primary antibody mouse anti-SMA1 (1:200, Invitrogen) for 2 h at room temperature. After these buffer washes, the samples were incubated with the respective secondary antibodies conjugated with Alexa Fluor 555 (donkey anti-mouse, 1:2000, Invitrogen) for 1 h. The samples were buffer-washed, mounted with Prolong Gold antifader reagent with DAPI (Invitrogen) for nuclear contrast staining and visualized by fluorescence microscopy (Leica DM6000B microscope, Leica Microsystems GmbH) and Diskus Viewer 4.8 (Hilgers Technisches Büro e. K., Königswinter, Germany). Experiments were conducted in triplicate for 5 donors.

### 4.5. Real-Time Polymerase Chain Reaction (RT-PCR)

The cells were seeded at 9000 cells/1.8 cm^2^ on collagen-I-coated 24-well plates (Corning, New York, NY, USA) and incubated in different media (Table 2) for 48 h at 37 °C. The total RNA from cultured cells was extracted using RNeasy MiniKit (Qiagen, Hilden, Germany), according to the manufacturer’s protocol. Reverse transcription was carried out with the Reverse Transcription System (Promega, Madison, WI, USA). Alterations in gene expression were analyzed by quantitative real-time PCR (RT-PCR) using the LightCycler FastStart DNA Master SYBR Green I kit (Roche) with the LightCycler 1.2 (Roche). The samples were taken in duplicate using the following primers (Supplementary Table S1): glyceraldehyde-3-phosphate dehydrogenase (GAPDH), α-smooth muscle actin (SMA), lumican (LUM) and aldehyde dehydrogenase family 3 member A1 (ALDH3A1). Relative fold changes in gene expression were analyzed using the comparative CT (2^−ΔΔCT^) method for 6 different donors [76]. Relative fold changes were calculated in comparison to the control group.

### 4.6. Statistical Analysis

All data are expressed as mean ± standard deviation (SD). Statistical analyses were performed with SPSS version 22.0 (IBM, Chicago, IL, USA). The Mann–Whitney U test or Wilcoxon rank-sum test were used to compare cell numbers and gene ratios. A *p* value of ≤0.05 was considered statistically significant.

## Figures and Tables

**Table 1:** Cell count and real-time polymerase chain reaction (RT-PCR) results for aldehyde dehydrogenase family 3 member A1 (ALDH3A1), lumican (LUM) and α-smooth muscle actin (SMA) in activated corneal stromal keratocytes (aCSK) after incubation for 48 h in media containing the indicated concentrations of the antifibrotic molecules (AFM) caffeine, curcumin and pirfenidone, with and without the profibrotic cytokine 10 ng/mL TGF-β1. Relative fold changes were calculated in comparison to the control group. Significant differences between groups are indicated by * *p* ≤ 0.05, ** *p* ≤ 0.01 and *** *p* ≤ 0.001.

	SMA (Fold Change)	LUM (Fold Change)	ALDH3A1 (Fold Change)	Cell Count/mm^2^
basal medium = control	1	1	1	55.3 ± 4.4
control + TGF-β1	10.16 ± 3.10 ***	0.61 ± 0.07 ***	0.02 ± 0.02 ***	57.2 ± 6.3
caffeine 50 µM	0.99 ± 0.24	0.95 ± 0.10	1.09 ± 0.22	52.6 ± 11.8
caffeine 50 µM + TGF-β1	9.77 ±2.58	0.73 ± 0.20	0.02 ± 0.01	54.0 ± 8.1
caffeine 100 µM	0.93 ± 0.38	1.00 ± 0.20	1.06 ± 0.22	52.4 ± 8.5
caffeine 100 µM + TGF-β1	6.06 ± 1.03 *	0.59 ± 0.14	0.01 ± 0.01	54.2 ± 9.1
caffeine 500 µM	1.10 ± 0.47	0.75 ± 0.14 ***	1.19 ± 0.22	42.8 ± 15.0 *
caffeine 500 µM + TGF-β1	10.23 ± 4.50	0.64 ± 0.16	0.01 ± 0.01	45.7 ± 8.1 *
curcumin 20 µM	0.93 ± 0.16	0.84 ± 0.25	0.94 ± 0.25	46.4 ± 12.7
curcumin 20 µM + TGF-β1	3.96 ± 0.17 **	0.52 ± 0.19	0.01 ± 0.01	48.7 ± 9.5
curcumin 50 µM	1.02 ± 0.30	0.78 ± 0.18 **	1.12 ± 0.18	21.2 ± 4.1 ***
curcumin 50 µM + TGF-β1	2.94 ± 0.76 **	0.55 ± 0.14	0.01 ± 0.01	20.5 ± 4.1 ***
pirfenidone 1.1 nM	1.09 ± 0.14	1.34 ± 0.15 ***	0.48 ± 0.06 ***	53.3 ± 7.0
pirfenidone 1.1 nM + TGF-β1	6.88 ± 2.32 *	0.55 ± 0.09	0.01 ± 0.01	54.2 ± 6.3
pirfenidone 2.2 nM	1.10 ± 0.13	1.44 ± 0.34 **	0.32 ± 0.09 ***	41.9 ± 7.7 ***
pirfenidone 2.2 nM + TGF-β1	7.92 ± 1.89	0.45 ± 0.07 **	0.01 ± 0.01	42.4 ± 5.1 ***

**Table 2:** Medium composition for testing the effects of the antifibrotic molecules (AFM) caffeine, curcumin and pirfenidone in activated primary human corneal stromal keratocytes (aCSK).

basal medium = control	DMEM/Ham’s F12 (Merck) +1% MEM nonessential amino acids (Gibco) +0.8% Penicillin-Streptomycin (Sigma-Aldrich) +1% Amphotericin B (Sigma-Aldrich) +1% MEM Eagle’s Vitamin Mix (Merck) +1 mM L-ascorbate 2-phosphate (Sigma-Aldrich) +10 µM ROCK-inhibitor (AdooQ Bioscience) +10 ng/mL Insulin-like growth factor (Gibco) +0.5% FBS (Panbiotech)
control + TGF-β1	control + 10 ng/mL TGF-β1 (human TGF-β1, PeproTech, Cranbury, NJ, USA)
caffeine 50 µM	control + 50 µM caffeine (Sigma-Aldrich)
caffeine 50 µM + TGF-β1	control + 50 µM caffeine + 10 ng/mL TGF-β1
caffeine 100 µM	control + 100 µM caffein
caffeine 100 µM + TGF-β1	control + 100 µM caffeine + 10 ng/mL TGF-β1
caffeine 500 µM	control + 500 µM caffeine
caffeine 500 µM + TGF-β1	control + 500 µM caffeine + 10 ng/mL TGF-β1
curcumin 20 µM	control + 20 µM curcumin (Sigma-Aldrich)
curcumin 20 µM + TGF-β1	control + 20 µM curcumin + 10 ng/mL TGF-β1
curcumin 50 µM	control + 50 µM curcumi
curcumin 50 µM + TGF-β1	control + 50 µM curcumin + 10 ng/mL TGF-β1
pirfenidone 1.1 nM	control + 200 µg/mL pirfenidone (Sigma-Aldrich)
pirfenidone 1.1 nM + TGF-β1	control + 200 µg/mL pirfenidone + 10 ng/mL TGF-β1
pirfenidone 2.2 nM	control + 400 µg/mL pirfenidone
pirfenidone 2.2 nM + TGF-β1	control + 400 µg/mL pirfenidone + 10 ng/mL TGF-β1

## Data Availability

All data can be requested form the corresponding author.

## Supplementary Materials

The following supporting information can be downloaded at: https://www.mdpi.com/article/10.3390/ijms24021461/s1.

## References

[1] Fuest M., Yam G.H., Peh G.S., Mehta J.S. (2016). Advances in corneal cell therapy. Regen. Med..

[2] Fuest M., Ang M., Htoon H.M., Tan D., Mehta J.S. (2017). Long-term Visual Outcomes Comparing Descemet Stripping Automated Endothelial Keratoplasty and Penetrating Keratoplasty. Am. J. Ophthalmol..

[3] Andresen J.L., Ledet T., Ehlers N. (1997). Keratocyte migration and peptide growth factors: The effect of PDGF, bFGF, EGF, IGF-I, aFGF and TGF-beta on human keratocyte migration in a collagen gel. Curr. Eye Res..

[4] Yam G.H., Yusoff N.Z., Kadaba A., Tian D., Myint H.H., Beuerman R.W., Zhou L., Mehta J.S. (2015). Ex Vivo Propagation of Human Corneal Stromal “Activated Keratocytes” for Tissue Engineering. Cell Transplant..

[5] Yam G.H., Teo E.P., Setiawan M., Lovatt M.J., Yusoff N., Fuest M., Goh B.T., Mehta J.S. (2018). Postnatal periodontal ligament as a novel adult stem cell source for regenerative corneal cell therapy. J. Cell. Mol. Med..

[6] Espana E.M., Kawakita T., Liu C.Y., Tseng S.C. (2004). CD-34 expression by cultured human keratocytes is downregulated during myofibroblast differentiation induced by TGF-beta1. Investig. Ophthalmol. Vis. Sci..

[7] Grobe G.M., Reichl S. (2013). Characterization of vitamin C-induced cell sheets formed from primary and immortalized human corneal stromal cells for tissue engineering applications. Cells Tissues Organs.

[8] Guo X., Hutcheon A.E., Melotti S.A., Zieske J.D., Trinkaus-Randall V., Ruberti J.W. (2007). Morphologic characterization of organized extracellular matrix deposition by ascorbic acid-stimulated human corneal fibroblasts. Investig. Ophthalmol. Vis. Sci..

[9] Pei Y., Sherry D.M., McDermott A.M. (2004). Thy-1 distinguishes human corneal fibroblasts and myofibroblasts from keratocytes. Exp. Eye Res..

[10] Jester J.V., Huang J., Fisher S., Spiekerman J., Chang J.H., Wright W.E., Shay J.W. (2003). Myofibroblast differentiation of normal human keratocytes and hTERT, extended-life human corneal fibroblasts. Investig. Ophthalmol. Vis. Sci..

[11] Jester J.V., Brown D., Pappa A., Vasiliou V. (2012). Myofibroblast differentiation modulates keratocyte crystallin protein expression, concentration, and cellular light scattering. Investig. Ophthalmol. Vis. Sci..

[12] Binte M.Y.N.Z., Riau A.K., Yam G.H.F., Binte Halim N.S.H., Mehta J.S. (2022). Isolation and Propagation of Human Corneal Stromal Keratocytes for Tissue Engineering and Cell Therapy. Cells.

[13] Seidelmann N., Duarte Campos D.F., Rohde M., Johnen S., Salla S., Yam G.H., Mehta J.S., Walter P., Fuest M. (2021). Human platelet lysate as a replacement for fetal bovine serum in human corneal stromal keratocyte and fibroblast culture. J. Cell. Mol. Med..

[14] Arshinoff S.A., Mills M.D., Haber S. (1996). Pharmacotherapy of photorefractive keratectomy. J. Cataract. Refract. Surg..

[15] Jester J.V., Nien C.J., Vasiliou V., Brown D.J. (2012). Quiescent keratocytes fail to repair MMC induced DNA damage leading to the long-term inhibition of myofibroblast differentiation and wound healing. Mol. Vis..

[16] Møller-Pedersen T., Cavanagh H.D., Petroll W.M., Jester J.V. (1998). Neutralizing antibody to TGFbeta modulates stromal fibrosis but not regression of photoablative effect following PRK. Curr. Eye Res..

[17] Bühren J., Nagy L., Swanton J.N., Kenner S., MacRae S., Phipps R.P., Huxlin K.R. (2009). Optical effects of anti-TGFbeta treatment after photorefractive keratectomy in a cat model. Investig. Ophthalmol. Vis. Sci..

[18] Saika S., Yamanaka O., Okada Y., Miyamoto T., Kitano A., Flanders K.C., Ohnishi Y., Nakajima Y., Kao W.W., Ikeda K. (2007). Effect of overexpression of PPARgamma on the healing process of corneal alkali burn in mice. Am. J. Physiol. Cell Physiol..

[19] Kuriyan A.E., Lehmann G.M., Kulkarni A.A., Woeller C.F., Feldon S.E., Hindman H.B., Sime P.J., Huxlin K.R., Phipps R.P. (2012). Electrophilic PPARgamma ligands inhibit corneal fibroblast to myofibroblast differentiation in vitro: A potentially novel therapy for corneal scarring. Exp. Eye Res..

[20] Berkowski W.M., Gibson D.J., Seo S., Proietto L.R., Whitley R.D., Schultz G.S., Plummer C.E. (2018). Assessment of Topical Therapies for Improving the Optical Clarity Following Stromal Wounding in a Novel Ex Vivo Canine Cornea Model. Investig. Ophthalmol. Vis. Sci..

[21] Tatler A.L., Barnes J., Habgood A., Goodwin A., McAnulty R.J., Jenkins G. (2016). Caffeine inhibits TGFβ activation in epithelial cells, interrupts fibroblast responses to TGFβ, and reduces established fibrosis in ex vivo precision-cut lung slices. Thorax.

[22] Chao S.C., Hu D.N., Roberts J., Shen X., Lee C.Y., Nien C.W., Lin H.Y. (2017). Inhibition effect of curcumin on UVB-induced secretion of pro-inflammatory cytokines from corneal limbus epithelial cells. Int. J. Ophthalmol..

[23] Jiang N., Ma M., Li Y., Su T., Zhou X.Z., Ye L., Yuan Q., Zhu P., Min Y., Shi W. (2018). The role of pirfenidone in alkali burn rat cornea. Int. Immunopharmacol..

[24] Wilson S.E., He Y.G., Weng J., Li Q., McDowall A.W., Vital M., Chwang E.L. (1996). Epithelial injury induces keratocyte apoptosis: Hypothesized role for the interleukin-1 system in the modulation of corneal tissue organization and wound healing. Exp. Eye Res..

[25] Wilson S.E., Mohan R.R., Hong J.W., Lee J.S., Choi R., Mohan R.R. (2001). The wound healing response after laser in situ keratomileusis and photorefractive keratectomy: Elusive control of biological variability and effect on custom laser vision correction. Arch. Ophthalmol..

[26] Huxlin K.R., Hindman H.B., Jeon K.I., Bühren J., MacRae S., DeMagistris M., Ciufo D., Sime P.J., Phipps R.P. (2013). Topical rosiglitazone is an effective anti-scarring agent in the cornea. PLoS ONE.

[27] Wilson S.E., Li Q., Weng J., Barry-Lane P.A., Jester J.V., Liang Q., Wordinger R.J. (1996). The Fas-Fas ligand system and other modulators of apoptosis in the cornea. Investig. Ophthalmol. Vis. Sci..

[28] Huang P.T., Nelson L.R., Bourne W.M. (1989). The morphology and function of healing cat corneal endothelium. Investig. Ophthalmol. Vis. Sci..

[29] Erie J.C., Patel S.V., McLaren J.W., Maguire L.J., Ramirez M., Bourne W.M. (1999). Keratocyte density in vivo after photorefractive keratectomy in humans. Trans. Am. Ophthalmol. Soc..

[30] Welsh E.J., Bara A., Barley E., Cates C.J. (2010). Caffeine for asthma. Cochrane Database Syst. Rev..

[31] Modi A.A., Feld J.J., Park Y., Kleiner D.E., Everhart J.E., Liang T.J., Hoofnagle J.H. (2010). Increased caffeine consumption is associated with reduced hepatic fibrosis. Hepatology.

[32] Furtado K.S., Polletini J., Dias M.C., Rodrigues M.A., Barbisan L.F. (2014). Prevention of rat liver fibrosis and carcinogenesis by coffee and caffeine. Food Chem. Toxicol..

[33] Gordillo-Bastidas D., Oceguera-Contreras E., Salazar-Montes A., Gonzalez-Cuevas J., Hernandez-Ortega L.D., Armendariz-Borunda J. (2013). Nrf2 and Snail-1 in the prevention of experimental liver fibrosis by caffeine. World J. Gastroenterol..

[34] Arauz J., Zarco N., Segovia J., Shibayama M., Tsutsumi V., Muriel P. (2014). Caffeine prevents experimental liver fibrosis by blocking the expression of TGF-beta. Eur. J. Gastroenterol. Hepatol..

[35] Wang H., Guan W., Yang W., Wang Q., Zhao H., Yang F., Lv X., Li J. (2014). Caffeine inhibits the activation of hepatic stellate cells induced by acetaldehyde via adenosine A2A receptor mediated by the cAMP/PKA/SRC/ERK1/2/P38 MAPK signal pathway. PLoS ONE.

[36] Fehrholz M., Speer C.P., Kunzmann S. (2014). Caffeine and rolipram affect Smad signalling and TGF-beta1 stimulated CTGF and transgelin expression in lung epithelial cells. PLoS ONE.

[37] Kovacs E.M., Stegen J., Brouns F. (1998). Effect of caffeinated drinks on substrate metabolism, caffeine excretion, and performance. J. Appl. Physiol..

[38] Willson C. (2018). The clinical toxicology of caffeine: A review and case study. Toxicol. Rep..

[39] Wilson S.E. (2012). Corneal myofibroblast biology and pathobiology: Generation, persistence, and transparency. Exp. Eye Res..

[40] Jester J.V., Barry-Lane P.A., Cavanagh H.D., Petroll W.M. (1996). Induction of alpha-smooth muscle actin expression and myofibroblast transformation in cultured corneal keratocytes. Cornea.

[41] Radomska-Lesniewska D.M., Osiecka-Iwan A., Hyc A., Gozdz A., Dabrowska A.M., Skopinski P. (2019). Therapeutic potential of curcumin in eye diseases. Cent. Eur. J. Immunol..

[42] Schaffer M., Schaffer P.M., Zidan J., Sela G.B. (2011). Curcuma as a functional food in the control of cancer and inflammation. Curr. Opin. Clin. Nutr. Metab. Care.

[43] Panahi Y., Hosseini M.S., Khalili N., Naimi E., Simental-Mendia L.E., Majeed M., Sahebkar A. (2016). Effects of curcumin on serum cytokine concentrations in subjects with metabolic syndrome: A post-hoc analysis of a randomized controlled trial. Biomed. Pharmacother..

[44] Lin Y.G., Kunnumakkara A.B., Nair A., Merritt W.M., Han L.Y., Armaiz-Pena G.N., Kamat A.A., Spannuth W.A., Gershenson D.M., Lutgendorf S.K. (2007). Curcumin inhibits tumor growth and angiogenesis in ovarian carcinoma by targeting the nuclear factor-kappaB pathway. Clin. Cancer Res..

[45] Marchiani A., Rozzo C., Fadda A., Delogu G., Ruzza P. (2014). Curcumin and curcumin-like molecules: From spice to drugs. Curr. Med. Chem..

[46] Santibáñez J.F., Quintanilla M., Martínez J. (2000). Genistein and curcumin block TGF-beta 1-induced u-PA expression and migratory and invasive phenotype in mouse epidermal keratinocytes. Nutr. Cancer.

[47] Kim J.S., Choi J.S., Chung S.K. (2010). The effect of curcumin on corneal neovascularization in rabbit eyes. Curr. Eye Res..

[48] Bolger G.T., Licollari A., Tan A., Greil R., Vcelar B., Greil-Ressler S., Weiss L., Schönlieb C., Magnes T., Radl B. (2019). Pharmacokinetics of liposomal curcumin (Lipocurc™) infusion: Effect of co-medication in cancer patients and comparison with healthy individuals. Cancer Chemother. Pharmacol..

[49] Kanai M., Imaizumi A., Otsuka Y., Sasaki H., Hashiguchi M., Tsujiko K., Matsumoto S., Ishiguro H., Chiba T. (2012). Dose-escalation and pharmacokinetic study of nanoparticle curcumin, a potential anticancer agent with improved bioavailability, in healthy human volunteers. Cancer Chemother. Pharmacol..

[50] Hewlings S.J., Kalman D.S. (2017). Curcumin: A Review of Its Effects on Human Health. Foods.

[51] Lao C.D., Ruffin M.T.t., Normolle D., Heath D.D., Murray S.I., Bailey J.M., Boggs M.E., Crowell J., Rock C.L., Brenner D.E. (2006). Dose escalation of a curcuminoid formulation. BMC Complement. Altern. Med..

[52] Sharma R.A., Euden S.A., Platton S.L., Cooke D.N., Shafayat A., Hewitt H.R., Marczylo T.H., Morgan B., Hemingway D., Plummer S.M. (2004). Phase I clinical trial of oral curcumin: Biomarkers of systemic activity and compliance. Clin. Cancer Res..

[53] Lin C.C., Lin H.Y., Chi M.H., Shen C.M., Chen H.W., Yang W.J., Lee M.H. (2014). Preparation of curcumin microemulsions with food-grade soybean oil/lecithin and their cytotoxicity on the HepG2 cell line. Food Chem..

[54] Liu F., Bayliss G., Zhuang S. (2019). Application of nintedanib and other potential anti-fibrotic agents in fibrotic diseases. Clin. Sci..

[55] Taniguchi H., Ebina M., Kondoh Y., Ogura T., Azuma A., Suga M., Taguchi Y., Takahashi H., Nakata K., Sato A. (2010). Pirfenidone in idiopathic pulmonary fibrosis. Eur. Respir. J..

[56] Choi K., Lee K., Ryu S.W., Im M., Kook K.H., Choi C. (2012). Pirfenidone inhibits transforming growth factor-beta1-induced fibrogenesis by blocking nuclear translocation of Smads in human retinal pigment epithelial cell line ARPE-19. Mol. Vis..

[57] Sun G., Lin X., Zhong H., Yang Y., Qiu X., Ye C., Wu K., Yu M. (2011). Pharmacokinetics of pirfenidone after topical administration in rabbit eye. Mol. Vis..

[58] Macias-Barragan J., Sandoval-Rodriguez A., Navarro-Partida J., Armendariz-Borunda J. (2010). The multifaceted role of pirfenidone and its novel targets. Fibrogenesis Tissue Repair.

[59] Choi Y.H., Back K.O., Kim H.J., Lee S.Y., Kook K.H. (2013). Pirfenidone attenuates IL-1β-induced COX-2 and PGE2 production in orbital fibroblasts through suppression of NF-κB activity. Exp. Eye Res..

[60] Hewitson T.D., Kelynack K.J., Tait M.G., Martic M., Jones C.L., Margolin S.B., Becker G.J. (2001). Pirfenidone reduces in vitro rat renal fibroblast activation and mitogenesis. J. Nephrol..

[61] Kaur H., Chaurasia S.S., de Medeiros F.W., Agrawal V., Salomao M.Q., Singh N., Ambati B.K., Wilson S.E. (2009). Corneal stroma PDGF blockade and myofibroblast development. Exp. Eye Res..

[62] Singh V., Santhiago M.R., Barbosa F.L., Agrawal V., Singh N., Ambati B.K., Wilson S.E. (2011). Effect of TGFbeta and PDGF-B blockade on corneal myofibroblast development in mice. Exp. Eye Res..

[63] Kim W.J., Mohan R.R., Mohan R.R., Wilson S.E. (1999). Effect of PDGF, IL-1alpha, and BMP2/4 on corneal fibroblast chemotaxis: Expression of the platelet-derived growth factor system in the cornea. Investig. Ophthalmol. Vis. Sci..

[64] Lin X., Yu M., Wu K., Yuan H., Zhong H. (2009). Effects of pirfenidone on proliferation, migration, and collagen contraction of human Tenon’s fibroblasts in vitro. Investig. Ophthalmol. Vis. Sci..

[65] Jung K.I., Choi J.S., Kim H.K., Shin S.Y. (2012). Effects of an anti-transforming growth factor-beta agent (pirfenidone) on strabismus surgery in rabbits. Curr. Eye Res..

[66] Fink M.K., Giuliano E.A., Tandon A., Mohan R.R. (2015). Therapeutic potential of Pirfenidone for treating equine corneal scarring. Vet. Ophthalmol..

[67] Zhong H., Sun G., Lin X., Wu K., Yu M. (2011). Evaluation of pirfenidone as a new postoperative antiscarring agent in experimental glaucoma surgery. Investig. Ophthalmol. Vis. Sci..

[68] Singh V., Jaini R., Torricelli A.A., Santhiago M.R., Singh N., Ambati B.K., Wilson S.E. (2014). TGFβ and PDGF-B signaling blockade inhibits myofibroblast development from both bone marrow-derived and keratocyte-derived precursor cells in vivo. Exp. Eye Res..

[69] Chowdhury S., Guha R., Trivedi R., Kompella U.B., Konar A., Hazra S. (2013). Pirfenidone nanoparticles improve corneal wound healing and prevent scarring following alkali burn. PLoS ONE.

[70] Costabel U., Bendstrup E., Cottin V., Dewint P., Egan J.J., Ferguson J., Groves R., Hellström P.M., Kreuter M., Maher T.M. (2014). Pirfenidone in idiopathic pulmonary fibrosis: Expert panel discussion on the management of drug-related adverse events. Adv. Ther..

[71] Barranco-Garduño L.M., Buendía-Roldan I., Rodriguez J.J., González-Ramírez R., Cervantes-Nevárez A.N., Neri-Salvador J.C., Carrasco-Portugal M.D.C., Castañeda-Hernández G., Martinez-Espinosa K., Selman M. (2020). Pharmacokinetic evaluation of two pirfenidone formulations in patients with idiopathic pulmonary fibrosis and chronic hypersensitivity pneumonitis. Heliyon.

[72] Liu B., Li A., Wang H., Wang J., Zhai G., Ma H., Feng S., Liu L., Gao Y. (2020). Exploring the Key Genes and Pathways in the Formation of Corneal Scar Using Bioinformatics Analysis. Biomed. Res. Int..

[73] Wilson S.E. (2020). Corneal wound healing. Exp. Eye Res..

[74] Duarte Campos D.F., Rohde M., Ross M., Anvari P., Blaeser A., Vogt M., Panfil C., Yam G.H., Mehta J.S., Fischer H. (2019). Corneal bioprinting utilizing collagen-based bioinks and primary human keratocytes. J. Biomed. Mater. Res. A.

[75] Schindelin J., Arganda-Carreras I., Frise E., Kaynig V., Longair M., Pietzsch T., Preibisch S., Rueden C., Saalfeld S., Schmid B. (2012). Fiji: An open-source platform for biological-image analysis. Nat. Methods.

[76] Schmittgen T.D., Livak K.J. (2008). Analyzing real-time PCR data by the comparative C(T) method. Nat. Protoc..

