# Supplementary material for: Antifibrotic Effects of Caffeine, Curcumin and Pirfenidone in Primary Human Keratocytes
Source: Int J Mol Sci. 2023 Jan 11;24(2):1461. doi: 10.3390/ijms24021461 (PMC9862324; doi:10.3390/ijms24021461)
Supplement: Supplementary file 1 [file ijms-24-01461-s001.zip › ijms-2097417-supplementary.pdf]

Gene	Forward primer	Reverse primer
GAPDH, GenBank: NM_002046	5´-TGT GGT CAT GAG TCC TTC CA-3´	5´-CGA GAT CCC TCC AAA ATC AA-3´
SMA, GenBank: NM_001613.2	5´-CCC TGA AGT ACC CGA TAG AAC A-3´	5´-GGC AAC ACG AAG CTC ATT G-3´
LUM, GenBank: NM_002345.3	5´-CCT GGT TGA GCT GGA TCT GT-3´	5´-TGG TTT CTG AGA TGC GAT TG-3´
ALDH3A1, GenBank: NM_01135168.1	5´-CAT TGG CAC CTG GAA CTA CC-3´	5´-GGC TTG AGG ACC ACT GAG TT-3´

Supplemental Table S1 Primer sequences for gene expression analysis by RT-PCR in cultured human corneal stromal keratocytes (CSK).
